# Surface Electromyography Assessment-Guided Strengthening Exercises Considering Vastus Medialis and Vastus Lateralis Activation After Total Knee Arthroplasty: A Case Report

**DOI:** 10.7759/cureus.115966

**Published:** 2026-09-08

**Authors:** Masayoshi Sonoda, Hiroshi Katoh

**Affiliations:** 1 Department of Rehabilitation, Kyushu University of Nursing and Social Welfare, Tamana, JPN; 2 Department of Rehabilitation, Kumamoto Rehabilitation Hospital, Kikuyo, JPN; 3 Graduate School of Health Sciences, Yamagata Prefectural University of Health Sciences, Yamagata, JPN

**Keywords:** electromyography, japanese rehabilitation, strengthening exercises, total knee arthroplasty, vastus lateralis, vastus medialis

## Abstract

Reduced vastus medialis (VM) and vastus lateralis (VL) muscle activity after total knee arthroplasty (TKA) delays recovery of gait ability and activities of daily living. However, few studies have evaluated the muscle activity characteristics of the VM and VL using surface electromyography (SEMG) and used these findings to guide the selection of exercise tasks. Therefore, this case report describes the clinical course of a patient after an SEMG-guided exercise intervention in which exercise tasks eliciting the greatest muscle activation in the VM and VL were selected. A male patient in his 70s underwent TKA for right knee osteoarthritis. From postoperative day 1, range-of-motion exercises, strengthening exercises, gait training, and ADL training were initiated. From postoperative day 14, the muscle activity of the rectus femoris (RF), VM, and VL was assessed using SEMG during eight knee extension exercise tasks, and root mean square (RMS) values were calculated. The VM showed the highest RMS value during seated knee extension at 90° of knee flexion (SIT 90), whereas the VL showed the highest RMS value during maximal straight leg raise (SLR max). Therefore, these two exercise tasks were performed as strengthening exercises from postoperative days 14 to 56. The knee range of motion, extension lag, manual muscle testing, and pain intensity improved on postoperative day 56 compared with those on postoperative day 14. Additionally, the RMS values of the VM and VL and the VM/RF and VL/RF ratios increased, indicating a change in the RF-dominant muscle activity pattern. This case indicates that selecting exercise tasks according to the muscle activity characteristics of the VM and VL based on SEMG assessment may be a useful approach to quadriceps strengthening after TKA. Further controlled studies are needed to verify the effectiveness of this approach.

## Introduction

Total knee arthroplasty (TKA) is the most commonly performed surgical intervention for knee osteoarthritis, which is characterized by pain and impaired physical function, primarily due to articular cartilage degeneration and knee joint deformity. The number of procedures performed worldwide has been increasing [1]. In Japan, the number of TKA procedures is expected to continue to increase, underscoring the growing importance of postoperative rehabilitation following TKA [2].

Although TKA can reduce pain and improve physical function, recovery of gait ability and activities of daily living (ADL) requires a considerable amount of time [3]. Quadriceps muscle weakness significantly affects postoperative physical function and ADL performance [4,5]. Among the quadriceps muscles, the vastus medialis (VM) and vastus lateralis (VL) play crucial roles in knee joint stabilization, and impaired function of these muscles is associated with delayed recovery of gait ability and ADL [6]. Standardized quadriceps-strengthening exercises, such as quadriceps setting (Q setting) and straight leg raise (SLR), are frequently performed in clinical practice [7]. Recently, surface electromyography (SEMG), a method that noninvasively records electrical signals associated with muscle activity using electrodes placed on the skin surface, has been used to quantitatively evaluate muscle activity during exercise tasks [8]. However, studies on the effectiveness of interventions in which the muscle activity characteristics of individual quadriceps muscles are assessed using SEMG and exercise tasks are selected based on these findings in patients after TKA are limited.

Therefore, this case report aimed to describe the clinical course of a patient with delayed recovery of quadriceps function after TKA who underwent strengthening exercises selected based on SEMG assessment.

## Case presentation

A male patient in his 70s presented with right knee osteoarthritis. The patient’s height was 171 cm, body weight was 72 kg, and body mass index was 24.6 kg/m². Before presentation, he was independent in ADL and could ambulate independently without an assistive device. He was diagnosed with right knee osteoarthritis, with Kellgren-Lawrence classification grade III. He began experiencing right knee pain during work and ADL and presented to our hospital due to progressive worsening of pain. He had no medical history affecting muscle function or a history of outpatient rehabilitation. Right TKA was performed to reduce pain and improve ADL function.

Physical function was assessed on the day before surgery using a knee range-of-motion test (ROM-T), manual muscle testing (MMT), extension lag during SLR, pain assessment using the numerical rating scale (NRS), and the 10-m walk test at comfortable and maximum speeds. The ROM-T showed 130° of knee flexion and -15° of knee extension. The extension lag during SLR was 20°. MMT revealed grades 3 for knee flexion and 4 for knee extension. Knee extension muscle strength was assessed in the sitting position. The examiner applied moderate resistance to the knee at the maximum extension position that the patient could actively achieve. Because the patient was able to maintain this position against resistance, the MMT grade was determined to be 4. Pain was assessed using the NRS, and the pain intensity during knee flexion was 5/10. The 10-m walk time was 7.9 seconds at a comfortable speed and 7.3 seconds at the maximum speed. TKA was performed through a medial parapatellar approach using a cruciate-substituting (CS) Triathlon^®^ CS Total Knee System (Stryker, Kalamazoo, MI, USA). Physical therapy was initiated on postoperative day 1; however, the patient experienced difficulty performing active knee extension and SLR exercises and required assistance. A knee brace was also used during walking and transfer activities. Despite receiving standard postoperative physical therapy through postoperative day 13, marked quadriceps muscle weakness persisted, affecting gait and ADL training. Therefore, increasing the muscle activity of the VM and VL, which has been reported to be associated with the recovery of gait ability and ADL, was considered important. From postoperative day 14, after the polyurethane film dressing had been removed and SEMG sensors could be placed around the surgical wound, SEMG assessment of the VM and VL was performed, and the exercise tasks eliciting the highest muscle activity were selected. No weight-bearing restrictions were imposed, and no apparent complications, such as infection or deep vein thrombosis, were observed during the postoperative course.

Ethical considerations

This case report was prepared following the CARE guidelines [9]. This case report was approved by the Ethics Review Committee of Kumamoto Rehabilitation Hospital (approval number: 2026-16). The purpose and methods of the study were fully explained to the patient, and written informed consent was obtained.

Assessment and data analysis

Assessments were performed on the day before surgery and on days 14, 28, 42, and 56 (final assessment) after surgery. The preoperative assessments included ROM-T (knee flexion and extension), MMT (knee flexion and extension), extension lag during SLR, pain assessment using the NRS, and the 10-m walk test at comfortable and maximum speeds. From postoperative day 14 onward, ROM-T, MMT, extension lag during SLR, NRS, and SEMG assessments were performed (Figure 1).

**Figure 1:**
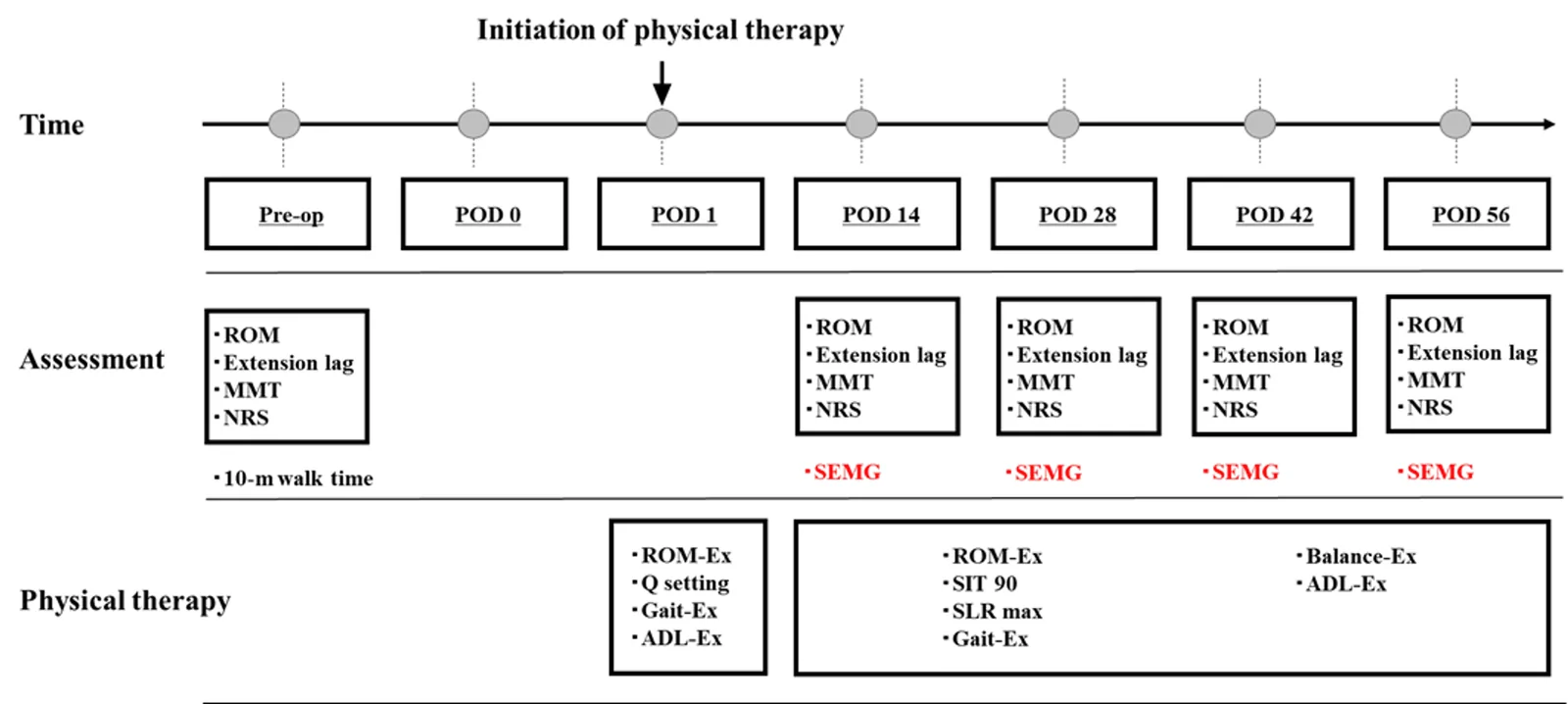
Timeline of the physical therapy intervention. ADL: activities of daily living; Ex: exercise; MMT: manual muscle testing; NRS: numerical rating scale; POD: postoperative day; Pre-op: preoperative (the day before surgery); ROM: range of motion; SEMG: surface electromyography; SIT 90: seated with 90° knee flexion; SLR max: straight leg raise with maximum

The exercise tasks used for SEMG assessment were selected based on those used in previous studies to evaluate the muscle activity characteristics of the quadriceps muscles, particularly the VM and VL. Specifically, the tasks included Q setting, in which the patient contracted the quadriceps muscle in the supine position with the knee in extension while pressing the popliteal fossa against the bed, and patella setting (P setting), performed in the same position as Q setting with the patient instructed to consciously pull the patella upward, knee extension from 30°, 60°, and 90° of knee flexion in the supine position (SUP 30, SUP 60, and SUP 90, respectively), isometric contraction at 40° of SLR (SLR 40), isometric contraction at the maximal SLR angle that could be actively achieved (SLR max), and knee extension from 90° of hip flexion and 90° of knee flexion in the sitting position (SIT 90) (Figure 2) [10-13]. 

**Figure 2:**
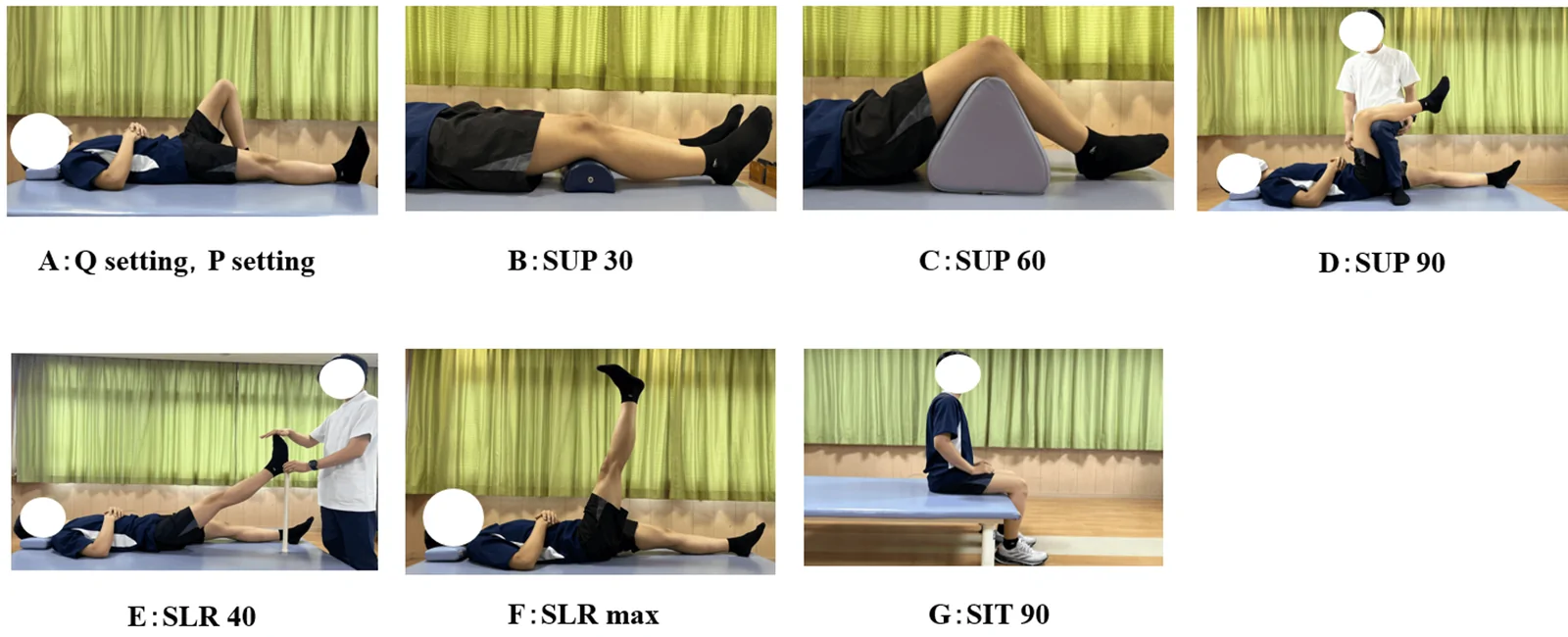
Exercise tasks. (A) Q setting and P setting, (B) SUP 30, (C) SUP 60, (D) SUP 90, (E) SLR 40, (F) SLR max, and (G) SIT 90. P setting: patella setting; Q setting: quadriceps setting; SIT 90: seated with 90° of knee flexion; SLR 40: straight leg raise with 40°; SLR max: straight leg raise with maximum; SUP 30: supine with 30° of knee flexion; SUP 60: supine with 60° of knee flexion; SUP 90: supine with 90° of knee flexion

Isometric contractions were maintained for three seconds in the starting position during the Q setting, P setting, SLR 40, and SLR max tasks. During the remaining tasks, the patient extended the knee joint as far as possible from the starting position and maintained an isometric contraction for three seconds at the maximal extension position that could be actively achieved. Each task was measured only once due to the potential for increased pain and fatigue. The central one-second interval of the recorded SEMG signals was used for analysis.

Muscle activity was assessed using the TS-MYO system (Trunk Solution, Tokyo, Japan). SEMG signals were acquired using TS-MYO sensors with electrodes placed over the RF, VM, and VL. The electrode placement sites were determined in accordance with the SENIAM guidelines (Figure 3) [14]. The acquired SEMG signals were processed using a dedicated application on an iPad with a 1-kHz sampling frequency and a 20-450 Hz band-pass filter. 

**Figure 3:**
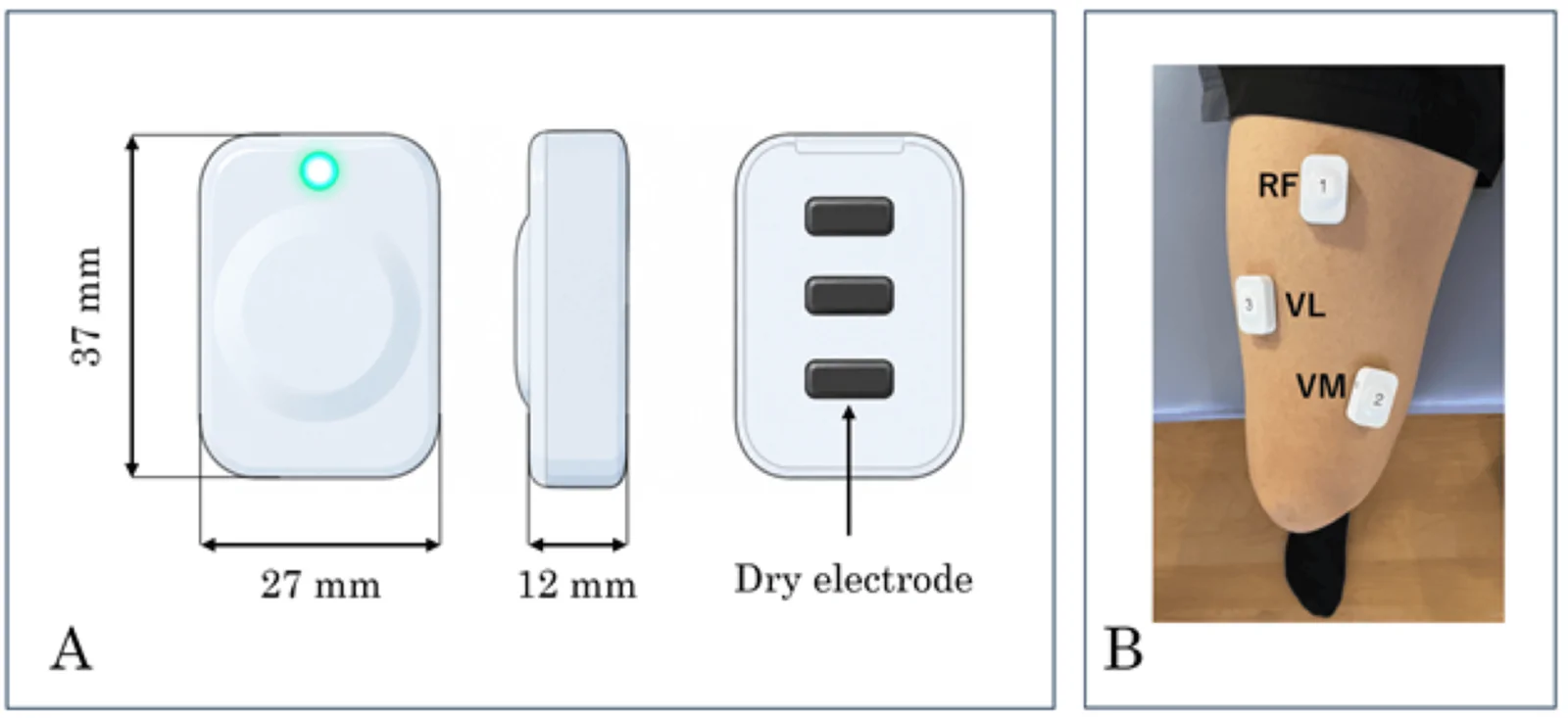
SEMG sensor and electrode placement sites. (A) SEMG sensor. The sensor measures 37 × 27 × 12 mm (length × width × thickness) and is equipped with dry electrodes. Data are wirelessly transmitted via Bluetooth. (B) Electrode placement sites. RF: halfway along the line connecting the anterior superior iliac spine and the superior border of the patella; VM: at the distal 1/5 of the line connecting the anterior superior iliac spine and the cleft at the anterior border of the medial collateral ligament; VL: at the distal 1/3 of the line connecting the anterior superior iliac spine and the lateral aspect of the patella. RF: rectus femoris; SEMG: surface electromyography; VM: vastus medialis; VL: vastus lateralis

Furthermore, the root mean square (RMS) was calculated using a 50-millisecond moving window with a one-millisecond step size and was used as an SEMG amplitude indicator [8]. RMS is frequently employed to quantify the amplitude of SEMG signals and is calculated by squaring the signal, calculating the mean, and then taking the square root. For each exercise task, the central one second of the three-second isometric contraction was manually selected, and the RMS value for this interval was calculated and used as the representative value (Figure 4). Furthermore, the VM/RF and VL/RF ratios were calculated to assess the relative muscle activity of the VM and VL compared with the RF. 

**Figure 4:**
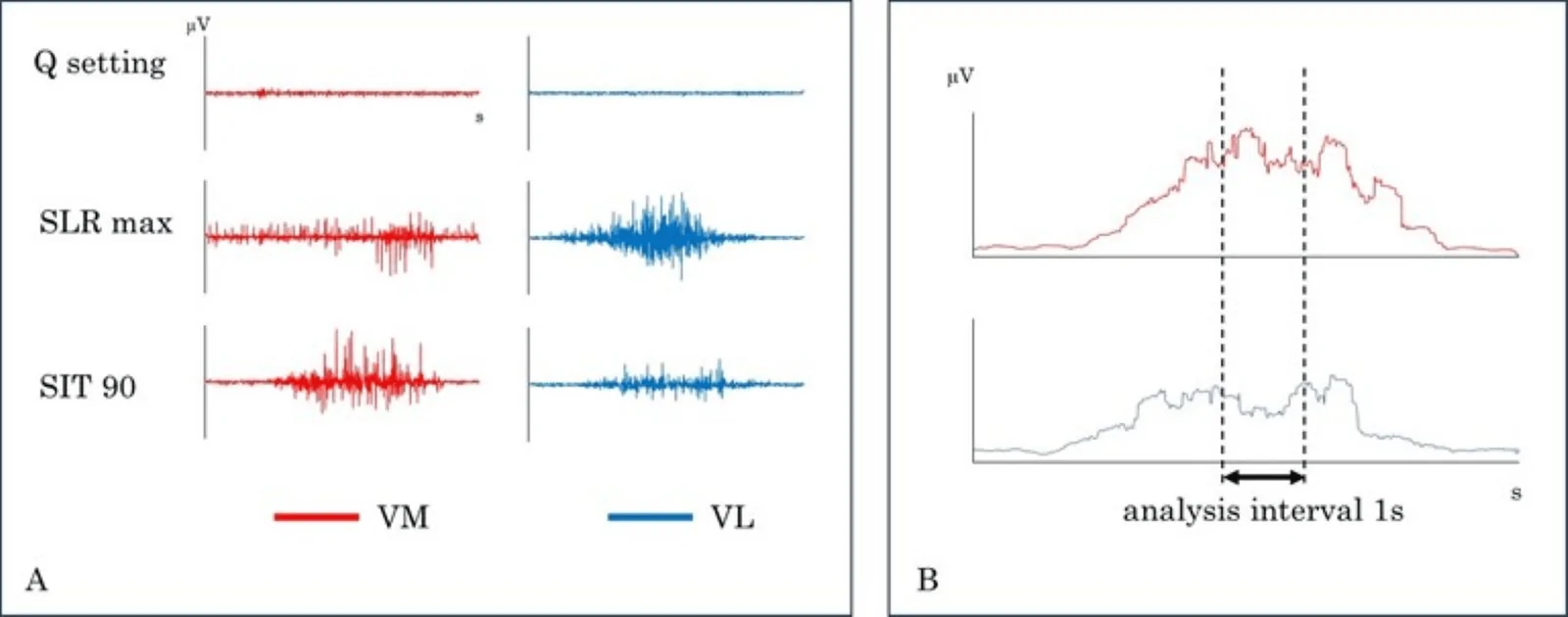
Muscle activity and RMS waveforms during the different exercise tasks. (A) VM and VL muscle activity waveforms during three exercise tasks (Q setting, SLR max, and SIT 90). The VM demonstrated the lowest muscle activity during Q setting and the highest during SIT 90. The VL demonstrated the lowest muscle activity during Q setting and the highest during SLR max. (B) RMS waveform during SIT 90. The central one second of the three-second isometric contraction was used as the analysis interval. Q setting: quadriceps setting; RMS: root mean square; SEMG: surface electromyography; SIT 90: seated with 90° knee flexion; SLR max: straight leg raise with maximum; VL: vastus lateralis; VM: vastus medialis

Physical therapy interventions

Physical therapy was performed twice daily, every day, with each session lasting 40 minutes. From postoperative days 1 to 13, the program focused on ROM exercises, strengthening exercises, gait training, and ADL training. For knee ROM exercises, active-assisted exercises were initiated on postoperative day 1, and passive exercises were added from postoperative day 2. For strengthening exercises, Q setting was initiated on postoperative day 1, and active-assisted knee extension exercises from a slightly flexed knee position were added from postoperative day 2. The number of repetitions was adjusted according to the patient’s pain and fatigue. Gait training was initiated in the parallel bars on postoperative day 3. A knee brace was used during gait training because of insufficient quadriceps muscle activity. From postoperative day 7, gait training with a walker was performed while the patient wore the knee brace. ADL training included wheelchair mobility and transfer training between the wheelchair and the bed or toilet, beginning on postoperative day 2.

From postoperative day 14 to postoperative day 56, the program focused on ROM exercises, strengthening exercises, gait training, balance training, and ADL training. From postoperative days 14 to 28, ROM exercises consisted of active-assisted and passive exercises. From postoperative day 28 onward, active exercises were added as pain and swelling decreased. From postoperative day 42 onward, active knee flexion and extension exercises performed in the sitting position and on the bed became the primary ROM exercises.

During this period, quadriceps-strengthening exercise tasks were selected based on SEMG assessment findings. Particularly, SIT 90 (Figure 2G), which showed the highest RMS value for the VM, and SLR max (Figure 2F), which showed the highest RMS value for the VL, were selected. SEMG assessments were performed at each assessment time point, and exercise tasks were re-evaluated. The VM continued to demonstrate the highest RMS value during SIT 90, and the VL continued to demonstrate the highest RMS value during SLR max on postoperative days 28, 42, and 56. Therefore, these exercise tasks were continued throughout the intervention period. Regarding the specific strengthening exercise procedures, during SIT 90, the patient was instructed to extend the knee joint as far as possible while ensuring that the posterior aspect of the thigh remained in contact with the bed, to maintain the maximal extension position for three seconds, and to slowly return to the starting position over three seconds. The patient was also instructed to maintain an upright trunk position to minimize compensatory movements involving the RF. The exercise consisted of 10 repetitions per set for five sets, with appropriate rest intervals between sets. During SLR max, the patient was instructed to maintain the maximally elevated position for three seconds and slowly lower the lower limb. During the exercise, the patient was instructed to raise the lower limb with the hip joint in a neutral position to minimize compensatory movements involving hip adduction and internal rotation. The patient had marked quadriceps muscle weakness and was unable to adequately control the lower limb during repeated SLR max, which could have resulted in rapid lowering of the limb. Therefore, considering safety and fatigue, the exercise was performed for three sets of 10 repetitions, which was the maximum number that could be performed safely. Additionally, the therapist positioned their hand to support the lower limb as needed to prevent sudden lowering. Both exercises were performed with 30-second rest periods between sets and within a pain-free range. Gait training consisted of walking with a walker without a knee brace from postoperative day 28, walking with a cane from postoperative day 42, and independent walking from postoperative day 56. Walking distance and speed were adjusted as needed according to pain and lower-limb fatigue. Balance training initiated on postoperative day 42 included single-leg standing on the operated side, maintaining a tandem stance, and weight-shifting exercises on a balance board. These exercises were adjusted as needed according to pain and lower-limb fatigue. ADL training initiated on postoperative day 42 primarily included stair ascent and descent, sitting on the floor, and household activities.

Results

Basic Physical Therapy Assessments

Table 1 shows the basic physical therapy assessment results at each assessment time point. On postoperative day 56, the knee flexion and extension ROM were 115° and 0°, respectively. The extension lag was 5°, the MMT scores for knee flexion and extension were 4, and the NRS score was 0. All assessment items on postoperative day 56 showed a trend toward improvement compared with those on postoperative day 14.

**Table 1:** Changes in basic physical therapy assessments. MMT: manual muscle testing; NRS: numerical rating scale; POD: postoperative day; Pre-op: preoperative (the day before surgery)

Measure	Pre-op	POD 14	POD 28	POD 42	POD 56
Knee flexion angle (°)	130	80	85	110	115
Knee extension angle (°)	-15	-15	-15	-5	0
Extension lag (°)	20	30	20	10	5
MMT (knee flexion)	3	2	3	4	4
MMT (knee extension)	4	2	3	4	4
NRS (knee flexion)	5	7	8	3	0
NRS (rest)	0	3	1	0	0
NRS (knee extension)	0	4	2	0	0

SEMG Assessments

The RMS values of the RF, VM, and VL for each exercise task are presented in Table 2 and the VM/RF and VL/RF ratios in Table 3. When comparing the RMS values of the RF, VM, and VL across the exercise tasks, the VM showed the highest value on postoperative day 14, when SEMG assessment was initiated, reaching 18.4 µV during SIT 90. Therefore, SIT 90 was selected as the exercise task for VM strengthening. In subsequent assessments, SIT 90 consistently elicited the highest value for the VM and was therefore continued as the exercise task until postoperative day 56. For the VL, SLR max showed the highest value on postoperative day 14, when SEMG assessment was initiated, reaching 15.9 µV. In subsequent assessments, SLR max consistently elicited the highest value for the VL and was therefore continued as the exercise task until postoperative day 56. On comparing postoperative days 14 and 56, the RMS values of the VM and VL exhibited an increasing trend across all exercise tasks. In contrast, the RF exhibited a decreasing trend during Q setting, P setting, SUP 60, and SLR max. The VM/RF and VL/RF ratios also showed an increasing trend across all exercise tasks. In particular, during Q and P settings, the RMS values of the VM and VL exhibited an increasing trend, whereas that of the RF showed a decreasing trend. Consequently, on postoperative day 56, both the VM/RF and VL/RF ratios during Q and P settings were ≥1, indicating greater muscle activity of the VM and VL compared with the RF than on postoperative day 14.

**Table 2:** RMS values of the RF, VM, and VL for each exercise task. Q setting: supine with quadriceps setting; P setting: supine with patella setting; SIT 90: seated with 90° of knee flexion; SLR 40: straight leg raise with 40°; SLR max: straight leg raise with maximum; SUP 30: supine with 30° of knee flexion; SUP 60: supine with 60° of knee flexion; SUP 90: supine with 90° of knee flexion POD: postoperative day; RF: rectus femoris; RMS: root mean square; VL: vastus lateralis; VM: vastus medialis

Exercise task	POD 14	POD 28	POD 42	POD 56
RF (μV)				
Q setting	3.74	2.43	2.47	2.4
P setting	3.9	2.5	2.79	2.34
SUP 30	12.8	22.6	23.1	17.9
SUP 60	26.8	24.8	24.3	22.6
SUP 90	20.1	26.3	34.1	25.3
SLR 40	15.5	23.9	54.2	29.2
SLR max	34.8	22.3	26	32.4
SIT 90	20.3	37.8	18.5	21.4
VM (μV)				
Q setting	3.08	2.7	3.64	4.69
P setting	2.86	3.89	9.2	5.49
SUP 30	4.8	4.23	8.54	6.93
SUP 60	5.84	7.71	5.56	10.6
SUP 90	7.6	10.4	26.7	22.8
SLR 40	3.58	8.88	5.35	7.84
SLR max	10.9	14.6	25.4	26.6
SIT 90	18.4	19.7	30.3	27.2
VL (μV)				
Q setting	2.4	2.9	3.08	2.62
P setting	2.22	4.88	5.31	6.37
SUP 30	4.55	3.86	8.26	10.6
SUP 60	4.96	14.3	8.84	13.2
SUP 90	5.02	18.8	14.4	19.1
SLR 40	3.47	21.1	15.6	10.4
SLR max	15.9	21.9	16.7	30.8
SIT 90	9.41	13.9	16.4	15

**Table 3:** VM/RF and VL/RF ratios for each exercise task. Q setting: supine with quadriceps setting; P setting: supine with patella setting; SIT 90: seated with 90° of knee flexion; SLR 40: straight leg raise with 40°; SLR max: straight leg raise with maximum; SUP 30: supine with 30° of knee flexion; SUP 60: supine with 60° of knee flexion; SUP 90: supine with 90° of knee flexion POD: postoperative day; RF: rectus femoris; VL: vastus lateralis; VM: vastus medialis

Exercise task	POD 14	POD 28	POD 42	POD 56
VM/RF				
Q setting	0.82	1.11	1.47	1.96
P setting	0.73	1.56	3.3	2.35
SUP 30	0.38	0.19	0.37	0.39
SUP 60	0.22	0.31	0.23	0.47
SUP 90	0.38	0.4	0.78	0.9
SLR 40	0.23	0.37	0.1	0.27
SLR max	0.31	0.65	0.98	0.82
SIT 90	0.91	0.52	1.64	1.27
VL/RF				
Q setting	0.64	1.19	1.25	1.09
P setting	0.57	1.95	1.9	2.72
SUP 30	0.36	0.17	0.36	0.59
SUP 60	0.19	0.58	0.36	0.58
SUP 90	0.25	0.71	0.42	0.75
SLR 40	0.22	0.88	0.29	0.36
SLR max	0.45	0.98	0.64	0.95
SIT 90	0.46	0.37	0.89	0.70

## Discussion

In this case, marked quadriceps muscle weakness was observed on postoperative day 14, which made gait and ADL training difficult. Therefore, muscle activity was assessed during knee extension exercises using SEMG on postoperative day 14. The results showed that the VM/RF and VL/RF ratios were <1 in all exercise tasks. The VM/RF and VL/RF ratios represent the relative muscle activity of the VM and VL compared with the RF, and values <1 indicate that the RF activity is relatively dominant compared with that of the VM and VL. Previous studies on healthy individuals have shown that the VM/RF and VL/RF ratios are generally ≥1 during knee extension exercises [15]. Therefore, this patient was considered to have an RF-dominant muscle activation pattern that differed from that observed in healthy individuals. Therefore, exercise tasks that demonstrated the highest muscle activity in the VM and VL were selected, and strengthening exercises focusing on these tasks were performed. By postoperative day 56, the RMS values of the VM and VL and the VM/RF and VL/RF ratios increased in all exercise tasks. However, because this was a single uncontrolled case, it cannot be determined whether these changes were attributable to the selection of exercise tasks based on SEMG assessment, conventional postoperative physical therapy, or natural recovery.

Quadriceps muscle weakness is common after TKA. Mizner et al. reported a marked reduction in quadriceps muscle strength within 1 month after TKA, approximately 62% compared with preoperative levels [16]. Consistent with previous studies, our case showed a marked reduction in quadriceps muscle strength on postoperative day 14, with an MMT grade of 2, and the patient had difficulty performing SLR. Postoperative quadriceps weakness after TKA is influenced more by muscle inactivity than by muscle atrophy [17]. Meier et al. reported that impaired muscle activation contributes to prolonged muscle weakness [18]. This phenomenon may be associated with arthrogenic muscle inhibition caused by surgical trauma, pain, and joint effusion. Abnormal afferent input from the joint may reduce α-motor neuron excitability, thereby suppressing quadriceps muscle activation [19].

In contrast, among the quadriceps muscles, the VM and VL are monoarticular muscles that function primarily in knee extension, whereas the RF is a biarticular muscle that also contributes to hip flexion. Therefore, during knee extension exercises after TKA, an activation pattern may occur in which the RF compensates for reduced activation of the VM and VL [20]. To improve gait ability and ADL performance, increasing the activation of the VM and VL, in addition to that of the RF, was considered important. Accordingly, from postoperative day 14 onward, exercise tasks eliciting the highest activation of the VM and VL were selected based on SEMG assessment. Specifically, SIT 90 was selected to maximize VM activation, whereas SLR max was selected to maximize VL activation. As these exercises involve knee extension with the hip in a relatively flexed position, the RF may have been placed in a shortened position, thereby facilitating relatively greater activation of the VM and VL.

The clinical significance of this case report lies in the use of SEMG to longitudinally assess the activation characteristics of individual quadriceps muscles and identify exercise tasks that elicited the greatest activation of the VM and VL, rather than routinely prescribing Q setting and SLR as quadriceps-strengthening exercises following TKA. Such an SEMG-guided rehabilitation is an approach that employs SEMG not simply as a measurement tool to evaluate the state of muscle activity but as an approach that utilizes the obtained muscle activity information to determine the treatment strategy. In this case, the muscle activities of the RF, VM, and VL during the eight exercise tasks were evaluated on postoperative day 14, and the exercise task that elicited the highest muscle activity in the VM and VL was selected as the muscle-strengthening exercise for each muscle. Furthermore, reassessments were conducted on postoperative days 28, 42, and 56, and the selected exercise tasks were continued following confirmation of the muscle activity characteristics at each time point. That is, in this case, SEMG was employed as an adjunctive decision-making tool to select exercise tasks based on the characteristics of the patients’ individual muscle activity and to determine whether to continue or modify them thereafter. Such an SEMG-based selection of exercise tasks may be a feasible method for individualizing quadriceps muscle-strengthening exercises following TKA according to the individual muscle activity characteristics of each patient.

This case report has several limitations. First, muscle activity was assessed using SEMG-derived RMS values and the VM/RF and VL/RF ratios, which do not directly measure muscle strength or hypertrophy. Second, the improvements observed in this case may have been influenced not only by the selection of exercise tasks based on SEMG assessment but also by natural recovery and conventional postoperative physical therapy. Therefore, the effects of the present intervention alone cannot be determined. Third, because gait analysis was not performed, the influence of changes in muscle activation patterns on gait biomechanics remains unclear. Fourth, because each exercise task was assessed only once using SEMG to minimize postoperative pain and fatigue, the reproducibility of the measurements could not be evaluated. Fifth, because this report describes a single case, the generalizability of the findings is limited, and the results should be interpreted with caution. Future studies involving larger samples and controlled study designs are needed to investigate the clinical usefulness of selecting exercise tasks based on SEMG assessment for improving muscle activation patterns and functional recovery after TKA.

## Conclusions

SIT 90 for the VM and SLR max for the VL elicited the highest muscle activity among the strengthening exercise tasks performed after TKA. During the intervention period, improvements in physical function were observed along with increases in the VM/RF and VL/RF ratios. However, it cannot be determined whether these changes were attributable to the selection of exercise tasks based on SEMG assessment. Future controlled studies are needed to determine whether SEMG-based exercise task selection is a useful approach to quadriceps strengthening after TKA.
